# Measuring Integrated Care – Methodological Reflections from Monitoring and Evaluation Process of the PHC Plus Pilot Program in Poland

**DOI:** 10.5334/ijic.6646

**Published:** 2023-04-03

**Authors:** Anna Kozieł, Marelize Gorgens, Mukesh Chawla, Anna Król-Jankowska, Aleksandra Kononiuk

**Affiliations:** 1 World Bank, BE; 2 World Bank, US; 3 World Bank, PL

**Keywords:** monitoring and evaluation, M&E, M&EF, theory of change, ToC, integrated care, measurement instruments, primary health care, PHC

## Abstract

**Introduction:**

Integrated care is an important strategy for increasing health system performance. Despite its growing significance, detailed evidence on the measurement properties of integrated care instruments remains vague and limited. This article aims to present the Monitoring and Evaluation Framework (M&EF) used in the pilot program of coordinated primary care in Poland. It can serve as an example of Monitoring and Evaluation (M&E) concept for the countries taking their first steps in the care integration implementation or establishing PHC reforms. This article belongs to the series of publications entitled: “Highway to hell or stairway to heaven – evaluating complex integrated care models – lessons from the Primary Healthcare Plus pilot program (PHC Plus) in Poland”.

**Methods:**

The M&EF of the PHC Plus was based on the Theory of Change within which a shift was to take place in the following 4 aspects: (1) change in health outcomes among participating patients; (2) change in health care experience among participating patients; (3) change in the fragmentation of care provided for chronic diseases among participating patients; (4) change in overall spending on health services for the patients in the PHC Plus facilities. Data for the M&EF of the PHC Plus came from two main sources: the National Health Fund (national insurer) database and the results survey questionnaires.

**Results:**

Based on the established M&EF of PHC Plus it was possible to monitor and evaluate the change in patients’ health outcomes, health experience and health literacy, fragmentation of care index, and overall spending on healthcare services offered in the pilot. Some of the analyzes planned could not be carried out due errors in data reporting systems, the lack of data of adequate quality or the pandemic. However, M&E implementation process provided many insightful information supporting broader PHC reform in Poland. Inclusive process of information and data sharing, discussions with the country health stakeholders and solid analytical background allowed for better informed policy making and scaling up the pilot.

**Conclusion:**

M&E process was put in place to help identify interventions, processes and approaches that could be scaled up and implemented at the country level. This approach resulted in broader understanding and acceptance of the proposed reforms in the PHC in Poland, following PHC Plus pilot implementation. Tools and approaches available and used to evaluate care integration in the PHC setting may not fully respond to the PHC and care integration system characteristic and country capacity. Therefore, revised M&E approach should be an integral part of the health policy interventions and further development of the PHC. Care integration specific measurement tools should be considered. Integration is a complex, multidimensional concept that requires measurement at multiple levels, including the patient level, the provider (professional) level, organizational and systems level. The M&E system of care integration requires use of multidimensional approach, complexed data systems, but also active sharing process of key findings. A phased approach is recommended to help researchers define clearly where they are in the research process and show progress on the outcomes’ achievement. Some simplification of the M&EF used in the PHC Plus is recommended to increase its sustainability without loosing key analytics.

## Introduction

Provision of accessible and safe primary care was recognized as a key priority for the World Health Organization (WHO) and its member states. Nevertheless, more than four decades after the Alma-Ata Declaration, the provision of primary health care remains inadequate, indicating that, in many parts of the world, including Poland, primary care has not received the priority it deserves. Instead of addressing the main health problems in the community through promotive, preventive, curative, and rehabilitative care, health system emphasis was put on inpatient care. This was partially driven by the historically designed system focusing on specialist and hospital care [1].

The existing model of PHC in Poland faces many challenges, with the most important ones being: (a) hospital-centric policies and funding; (b) inadequate quality of care; (c) unequal and, often, limited access to care for chronic care patients; (d) insufficient and constantly decreasing number of primary care personnel; uneven geographic distribution of PHC services; and (e) inadequate financing of PHC services [2].

Growing burden of non-communicable diseases (NCDs) and new patient’s expectations resulted in the need to shift from reactive PHC system to more patient-oriented, better integrated with other levels of care with new roles and responsibilities. Patient’s centric approach was implemented by the government of Poland who facilitated several initiatives aimed at care coordination, including ones at PHC level. The PHC in Poland is the part of the health care system that provides all eligible Poles with access to specific health services on an outpatient basis or, in medically justified cases, at the patient’s home. Almost entire financing of the PHC is capitation based. The PHC services in Poland cover: (i) the provision of disease prevention services and immunization; (ii) consultations on the treatment of diseases; (iii) laboratory, imaging, and non-imaging diagnostics (such as ECGs, X-rays, and ultrasounds); (iv) treatment in PHC facilities and in the patient’s home; (v) the prescription and management of medicine and medical devices; (vi) the verification of health conditions; (vii) the issuing of referrals to specialist clinics, hospitals, rehabilitation services, and others [3].

In May 2018, the National Health Fund (NHF) in Poland set up together with the Ministry of Health, the Primary Healthcare Plus pilot program (PHC Plus). The main objective of the pilot program was to test new interventions, services, processes, and care coordination in the PHC facilities. The pilot assumed that integrating care at the PHC level will help Poland to overcome some of the lingering unresolved issues in its health system and achieve its vision of service delivery reform, where the main point of contact and care is the PHC team. The pilot was also conceived as a way to step up the coordination of care in a bid to increase patient satisfaction, enhance the quality of care, increase access to services, overcome fragmentation in the delivery of care, enrich people’s care experiences, and improve care outcomes. The PHC Plus covered the basic scope of services delivered in PHC (mentioned above) and offered a range of additional services such as selected outpatient specialist care (OSC) and ambulatory physical therapy. In addition, PHC Plus facilities provided health check-ups, health education and a range of disease management programs (DMPs). PHC Plus pilot program offered also medical personnel capacity building towards broader range of competencies as well as expanding basic PHC team (GP, nurse, midwife) by adding care coordinator and well-managed access to selected specialists and physical therapists.

The health check-up preventive program offered in the PHC Plus pilot included health visits and a set of diagnostic tests aimed at stratifying the population into one of four groups: (i) healthy with no risk factors; (ii) healthy with no symptoms but some risk factors; (iii) chronic with no current symptoms and stable; and (iv) chronically ill with current symptoms and requiring stabilization. The pilot also set up DMPs for a range of chronic diseases and enrolled patients who qualified based on their health check-up or medical history. DMPs covered eleven chronic diseases: primary hypertension, type 2 diabetes, chronic coronary artery disease, permanent atrial fibrillation, chronic heart failure, asthma, chronic obstructive pulmonary disease (COPD), parenchymal goiter and thyroid nodule, hypothyroidism, peripheral osteoarthritis, and back pain. Patients in each disease-specific program received care that was coordinated at their PHC facility as well as specialist consultations, physiotherapy, and health education. Services provided under DMPs were delivered by broader medical teams including specialists, care coordinator and supervised by the core PHC personnel, usually a general practitioner (GP).

Several enabling tools were included in the PHC Plus pilot program to facilitate the provision of new model of care. The care coordinator in each facility managed the care pathway for each PHC Plus patient. Care coordinators could be either current PHC employees (administrative or medical) or a new staff member specially recruited for this purpose. The tasks of the coordinator included supporting primary care patients in their diagnostic and treatment process, ensuring better communication between PHC personnel, specialists, and the patient, informing patients about the next stages of treatment (including treatment beyond the PHC level of care), and supporting the organization of the treatment pathway, particularly for patients with chronic diseases.

The pilot was carried out in 47 preselected PHC facilities (71,000 participating patients) in Poland between July 2018 and September 2021. PHC facilities participating in the pilot were selected based on the call of proposals issued by the NHF. The pilot provided additional budget to each PHC Plus facility to finance the provision of the new services. Also, the provision of the new purchasing model, such as bundled payments (including fee-for-service) was introduced for the selected services within the PHC pilot. Concurrently to implementing the PHC Plus, the NHF in Poland requested support to develop and apply M&E system for the implementation of pilot model, including indicators, reporting formats, a database; advise on course corrections, as necessary; and after conclusion of the pilot, advise on its nationwide scale-up [4].

Monitoring and evaluation are powerful tools of policy making, providing insights and lessons learned for development of health systems. This article aims to present the Monitoring and Evaluation Framework (M&EF) developed and used for the PHC Plus pilot program in Poland to serve as an example of M&E concept of integrated care. The aim is also to present key lessons learned from the real life, complexed process, trying to bring closer the science of M&E, care integration and PHC development. Additional publications will present the key findings of the PHC Plus pilot program.

This article belongs to the series of publications entitled: “Highway to hell or stairway to heaven – evaluating complex integrated care models – lessons from the Primary Healthcare Plus pilot program (PHC Plus) in Poland”.

## Description of the M&E methodology

The M&E process for the PHC Plus pilot began with a thorough literature review on available integrated care monitoring and evaluation concepts and frameworks. Since significant information was already available, and several systematic reviews were conducted on the subject at that time (2018), we felt that there was no need to carry out another systematic review, but only to draw the most important conclusions from the existing reviews.

It was concluded that measuring and evaluating integrated care is a challenge, and there is no consensus on the key measurement elements, with multiple indicators and data source to use. The variety of M&E concepts reflects the significant conceptual diversity within the field, and most methods lack information regarding validity and reliability. At that time there were 24 different methods available to measure integrated healthcare delivery. Almost all methods were based on a theoretical model; however, some more rigorously than others. Structural and process aspects were often included in the measurement methods, while cultural aspects were rarely a part of the methods. Almost all of the available methods allowed evaluators to quantify their findings, but only a few to calculate sums and mean ranks of a combined measure of integration and none have been thoroughly validated across different settings [5,6].

More than 200 indicators to measure progress towards integrated care were already identified equally from the scientific and grey literature, including: well-known quantitative indicators (e.g., emergency admission rate, number of avoidable hospitalization rate) and qualitative indicators based on survey questionnaires (e.g., % of patients with positive experience of care, % of patients with increased health literacy). From these indicators many domains have been already defined, including access to care, care coordination, continuity of care, patient-centered care, community-based care, user experience and management/organization level. Moreover, these domains were additionally refined in the literature by more than 40 attributes [7].

Both the number of available approaches to M&E and multiple indicators meant that for the Poland’s specific M&E process at the PHC level, we had no other choice but to develop our own system that would best meet the needs of the M&E of the pilot itself. Available publications have guided M&E process of the PHC Plus pilot. The foundation of the M&EF was build based on the five assumptions:

1. Care integration is an incremental process that must be assessed as continuum and is anticipated to change over time [8];
2. Integration is a complex, multidimensional concept that requires measurement at multiple levels: the patient level (incl. patient-reported outcomes), the provider (professional) level, organizational and system levels (incl. workforce changes) [9,10,11];
3. The monitoring and evaluation of care integration requires use of different types of evidence and data sources [12];
4. Measuring continuum of care cascades in terms of service delivery, including management of chronic diseases, is a useful way to quantify integrated services [13,14,15];
5. Economic analysis of integrated care deviates significantly from standalone interventions with fixed and static cost-benefits analysis and are still in the process of development [10].

Literature review, implementation modalities and health system level constrain resulted in the preparation of the Theory of Change (ToC) approach for the M&E process.

Since the PHC Plus pilot project was designed to be very complex (involvement of both patients and caregivers in real life setting) the ToC was used not only to analyze the outcomes of an intervention, but also to serve as a source of evidence to support learning and accountability [16]. The implementation of the PHC Plus pilot M&E process resulted in the revision of the ToC. Most public health interventions are inherently complex, with multiple interacting components, delivered at multiple levels over time, making them difficult to evaluate using traditional experimental designs such as randomized controlled trials. Therefore, the ToC offered an alternative to improve the evaluation of complex health interventions.

For the purpose of the M&E of the PHC Plus, the ToC was defined as how a pilot program creates specific long-term outcomes through a logical sequence of intermediate outcomes. The ultimate success of any ToC lies in its ability to show progress on the outcomes’ achievement. The ToC helped to understand why a desired change (the PHC Plus pilot program) is expected to happen in a particular context. The evaluator tests a number of assumptions, designs data collection, and performs data analysis to follow the development of the assumptions [13]. This allowed to understand the relevance, effectiveness, efficiency, sustainability, and impact of the PHC Plus pilot program on the participating individuals and institutions.

At the start of the M&E process, ToC used a participatory approach by bringing together a range of stakeholders (health service planners, healthcare workers and service users) to develop a ToC map [17]. This took the form of a series of meetings, conferences, and workshops where the stakeholders agreed on the impact they want to achieve. In Poland this was done jointly with the NHF and MoH as a preparatory phase.

In addition, the Scientific Committee, together with broader group of health stakeholders and patient’s organizations, MoH and NHF identified the most important questions to be answered under the integrated care M&E process. These were:

1. Has there been any change in patient’s health outcomes?
2. Has there been any change in patient’s experience?
3. Has there been any change in fragmentation in the provision of chronic care services?
4. Has there been any impact of care integration on costs?

The data needed to obtain answers to the above questions were to come both from the data held by the NHF and reported directly by PHC Plus service providers, and from the data and information obtained from the patients themselves. We also knew that we had to plan dedicated analysis for chronically ill patients (incl. care cascades and care fragmentation) and to measure economic aspects of the PHC plus model. The Table 1 below presents a list of indicators as well as data sources required for planned analysis.

**Table 1:** Outcomes and associated indicators.


PHC PLUS EXPECTED CHANGE (OUTCOME)	INDICATOR	DATA SOURCE

Change in health outcomes of patients		

Improvement in the health outcomes of patients enrolled in the PHC Plus model, as reported by patients	% of PHC Plus registered chronic patients who reported an improvement in their PROM score from baseline to the end of the PHC Plus implementation period	PROM tools for 3 selected chronic care conditions for which tools exist

	Average % reduction in care cascade gaps for the 5 most prominent PHC Plus services for which temporal change could be managed	Care cascade constructs using NHF and PHC facilities datasets

Improvement in the ability and confidence of patients enrolled in the PHC Plus model to manage the patient’s condition	% of PHC Plus registered chronic patients who reported an improvement in their health literacy score (HLS) from baseline to the end of the PHC Plus implementation period	HLS tool for use in integrated chronic care

Lower hospitalization rate/acute conditions rate of patients enrolled in the PHC Plus model	Number of hospitalizations and acute conditions for any of the 11 chronic care conditions per 100 000 PHC Plus registered chronic patients, in the last 12 months	PHC Plus IT system, routine data transferred to NHF (electronic patient records)

Change in patient experience with chronic care		

Patients receive a more coordinated range of chronic care services with less fragmentation, as reported through patient-reported experience measures	% of PHC Plus registered chronic patients who reported an improvement in their PREM score from baseline to the end of the PHC Plus implementation period	PREM tool customized for purpose of integrated chronic care model

	Average number of health services per patient	PHC Plus IT system, routine data transferred to NHF (electronic patient records)

	% of PHC Plus registered chronic patients who receive all minimum health services recommended to them	PHC Plus IT system, routine data transferred to NHF (electronic patient records)

Patients have shorter waiting times and report that they can more easily navigate their way through the system	Average waiting time per service received (across each of the 11 chronic diseases)	PHC Plus IT system, routine data transferred to NHF (electronic patient records)

Family members are actively involved in patient’s healthcare	% of PHC Plus registered chronic patients who report that they receive targeted support from family members	PREM tool customized for purpose of integrated chronic care model

Reduce fragmentation in the provision of chronic care and lower costs		

Lower cost per-patient contact of health service received	Cost per contact-visit in the PHC pilot facilities	PHC Plus IT system, routine data transferred to NHF (electronic patient records)

Fewer duplicate laboratory tests	% of registered PHC Plus registered chronic patients who receive a duplicate laboratory test in the last 12 months	PHC Plus IT system, routine data (electronic patient records)

Better coordinated care with IT tools to strengthen integration and coordination at the patients, healthcare provider and payer level functioning	% of PHCs who order laboratory tests and receive results electronically	PHC Plus IT system, routine data (electronic patient records)

	% of times that specialists could access a patient’s electronic record at a location other than the one where the patient was registered, using the Integrated Patient Information System	PHC Plus IT system, routine data (electronic patient records)

	% of PHCs who have participated in electronic ordering and receiving results imaging tests performed outside the institution	PHC Plus IT system, routine data (electronic patient records)

Extended screening, prophylactic, and chronic care services	Average number of contact visits to provide expanded scope of services per 1000 population in catchment area, by type of service	PHC Plus IT system, routine data (electronic patient records)


## Results

Data for the M&E indicators were obtained from two databases: the NHF database and surveys database. No other source of data was available for the purpose of M&E of PHC Plus pilot model in Poland. The data availability was, in most cases, sufficient.

NHF database was the source of data on all of the 47 participating PHC facilities during the implementation period, including: the number of signed consents (participating patients) and conducted health care check- ups, as well as information relating to the processes in DMPs, and cost data of patient treatment for all health services offered to pilot beneficiaries across the health system.

Survey database included data from: patient-reported outcomes measures (PROM), patient-reported experience measures (PREM), and a health literacy (HL) measures. All survey questionnaires were verified with a positive opinion by the Bioethical Committee to ensure patient safety and maintain the highest ethical standards and norms. The detailed list of the selected questionnaires used in the process of M&EF of PHC pilot together with their results is presented in the article which can be found in the series of publications entitled: “Highway to hell or stairway to heaven – evaluating complex integrated care models – lessons from the Primary Healthcare Plus pilot program (PHC Plus) in Poland”. Detailed evaluation conclusions are to be published as a separate article.

The M&E of the PHC Plus pilot program has revealed changes in the patient’s health status after they joined the pilot. Their health literacy level and patient’s experience also changed in the curse of the pilot implementation. The M&E found that utilization of the health services, for the patients covered with the pilot changed on all levels of care (PHC, specialist care and rehabilitation and hospital, in patient care).

The PHC Plus measured detailed costs associated with the pilot and regular care provided in the health system for all the patients participating in the pilot. Value and volume of services provided measured under the M&E process supported following analyses forecasting potential consequences of the scaling up process.

Conducted M&E process provided evidence and detailed insight to broader PHC reforms organized around the needs of the patients, enabling them to play an active role in their own care, and providing prevention, education, pro-active and high-quality disease management services.

One of the key elements of the M&E process were regular stakeholders meetings discussing key findings and lessons learned supporting consensus and capacity building amongst health community.

## Discussion

To develop and define the different phases of the M&E for the PHC Plus, the literature review of a functional M&E was established before implementation of the pilot. At that time, no unified recommended guideline for assessing care integration within primary care level existed. However, based on available at that time literature and experts’ knowledge it was possible to indicate crucial aspects that should be included in each planned M&E process of integrated care, including: understanding what type of change (outcome) is expected and based on that, develop adequate research questions and indicators; using different types of evidence and data sources (derived both from service providers and patients themselves); focusing on chronically ill patients as a way to quantify integrated services and last but not least understanding that the financial assessment of such a model of care is inherently difficult to carry out.

Despite a well-developed theoretical approach to the planned M&E process, many practical limitations occurred. Although the process was carried out by independent external entity and had good theoretical support (based on ToC), the implementation process revealed that many of the assumptions had to be modified and adjusted to the changing reality. Limitations were mostly coming from the fact, that the M&E process was on top of the regular PHC service delivery, with only few additional tools available (reporting systems, data bases, capacity building). Legal constrains, limited timelines and financing also influenced M&E implementation modalities.

The developed M&E system did not use the randomized controlled trial structure, which is commonly considered the most valuable method of assessing the effectiveness of an intervention. In 2017, the NHF announced the recruitment of PHC service providers for the PHC Plus pilot. For legal reasons, it was not possible to choose the providers randomly. Instead, the NHF defined the selection criteria, the pre-selection regulations, and the conditions that the service providers had to meet. Over 800 service providers applied to be included, but by the end of the pilot, the NHF had chosen only 47 institutions to implement the PHC Plus. Furthermore, the fact that patients could sign up for PHC Plus at any time, while useful for implementation, made it difficult to develop a robust and consistent methodology for the evaluation. Thus, the observation period was short for some patients, and the evaluation had to be restricted to chronically ill patients who had been in a DMP for at least 12 months. The analyses conducted using the NHF databases were mainly aimed at detecting the quantitative implementation of the pilot at certain periods of time, mostly in terms of the number, cost, and types of health services provided by the PHC Plus facilities. External evaluators did not have the influence on the quality of the data provided. The quality of the NHF data on health service use was variable, and a large proportion of service utilization and costs data was missing from the datasets. This included data on PHC services, PHC financing, and emergency care financing. Nor was there any possibility of acquiring data on private health care provision of the patient’s supported by the PHC Plus pilot, or on over-the-counter medications used by the participating patients. Another obstacle was the quality of data reported by the providers, resulting in questionable ICD10 codes assigned to services. Also, because of GDPR regulations, limited data were available on the control group as there was no way to acquire their consent to access their service use data. Lastly, the COVID-19 pandemic put a big strain on the reliability of the NHF data from March 2020 until the end of the pilot.

Obstacles were also encountered in the context of data collection and analysis based on patient-reported metrics (survey database). Although M&EF assumed the use of various types of questionnaires, not all of them turned out to be usable. Some of the questionnaires recommended in the literature required the purchase of very expensive licenses, which limited their availability. Most of the integrated care questionaries found in the literature focused also on other levels of care (in-patient type of care in most cases) providing limited insight into the PHC monitoring process. Some questionnaires, although available for free, required translation or linguistic and cultural validation. Therefore, preparing questionnaires ready for use in the evaluation of PHC Plus was not only time-consuming, but also required the involvement of other experts depending on the scope of the evaluation or disease investigated, including specialists in the field of cultural research and translators.

Moreover, data on patient-reported measures collected through surveys were carried out by an external company, which was selected by the NHF in 2019 in an open-bid competition. Therefore, again the evaluators had no influence on the quality of the obtained data. The surveys were carried out in both PHC Plus and PHC control group facilities. The number of PHC Plus patients required to participate in the questionnaires was statistically matched and randomly distributed among all facilities. However, during the first round of the survey, three specific challenges were encountered in the selection of patients: (i) there were not enough patients in PHC Plus facilities who suffered from selected chronic diseases, such as asthma/COPD (fewer than 500 patients); (ii) there were not enough men in certain age groups with a specific disease in the selected PHC Plus facilities; and (iii) the first case of COVID-19 was detected in Poland. This led to a major reduction in the sample size. M&E process was conducted partially during COVID-19 pandemic which limited some of the data collection.

Despite the difficulties with the access and quality of data used for the planned by us M&E system, many valuable analyzes recommended in the literature and by the experts were successfully carried out. Based on available data it was possible to measure recommended in the literature the continuum or “cascade” of care – a useful way to quantify access to health services, their coverage and quality, and patients’ adherence to treatment throughout the sequential stages of care required to achieve a successful treatment outcome. The cascade of care involved four stages in a PHC Plus patient’s health care pathway: (i) screening, meaning whether the patient had been diagnosed with a health condition; (ii) diagnosis and treatment, meaning whether the patient had received appropriate health care; (iii) monitoring and maintenance, meaning whether the patient was adhering to the required care regimen; and (iv) outcome, meaning whether the patient’s disease was under control in the end of the pilot program. Since a failure at any stage of the cascade precludes success in the next stage, the evaluation measured each cascade step to identify any bottlenecks and solutions for overcoming them. Three selected care cascades were measured during the M&E of the PHC Plus pilot: (i) the health check-up cascade; (ii) the type 2 diabetes cascade; and (iii) the cardiology diseases cascade. Also, fragmentation of care index (FCI) was measured in order to analyze the dispersion of patient care. It measured the extent to which care fragmentation (i.e., number of different providers visited by each patient) had reduced for patients with selected chronic conditions after they entered a DMP.

The results of the surveys (incl. PROMs, PREMs and HLs) were also analyzed despite reduction of the sample size. With the use of score value ranging from 0 to 100, we could monitor and evaluate the change in patient’s perceived health outcomes, experience, and literacy. Apart from the basic cost analysis, it was also possible to conduct cost-utility analysis (CUA) based on results derived from one of the patients’ questionnaire we used, namely EQ-5D-5L questionnaire. CUA is an economic analysis in which the incremental cost of a program was compared to the incremental health improvement expressed in quality adjusted life years (QALYs), which are a measure of the value of health outcomes in terms of one year of life in full health.

All analyzes conducted as a part of the M&E process were consulted on an ongoing basis with program implementers, health facilities, health practitioners, and the results were provided on an ongoing basis in the form of written reports, workshops, and interactive dashboards (data visualization). Time frame and method of reporting were agreed with the contractor of the pilot before its commencement.

The timeline of the M&E process was three years, from the selection of the PHC facilities to the final report from the M&E. During this time regular, quarterly data collection had to be in place, two rounds of surveys conducted, and conclusions drawn. This time period may not have been sufficient to learn more on the patient’s outcomes or experience particularly. In reality, long term evaluation processes are more feasible to be applied in the academic setting rather than real health services organization.

## Conclusions

Integrated care can be an important strategy to increase health system performance, improving patient’s satisfaction or quality of care but despite its growing significance, robust evidence on system level, real lifecare integration measure needs to be developed further.

Health systems should include M&E tools, data collection systems and increase analytical capacity to conduct better evaluation of the new policy interventions. Ad hoc or even well-designed attempts of M&E may not always provide sufficient information on the interventions impact but can serve as a significant insight to the evidence-based policy making. M&E process should be imbedded and financed as a regular health system function. This area is beset with complexity that seems to emerge from: uncertainty about the nature of the integrated models and how they work, potential lack of agreement on the main objectives of integration, quality of data, inter-organizational relationships or leadership and limited evaluation tools designed for the PHC. However, “this complexity is not a reason not to evaluate but it does mean that well-designed approaches are required” [18].

While adopting the M&E of integrated care was by no means straightforward, it was certainly a step forward, presenting Poland’s efforts towards integrated primary care.
